# Mortality and Generalizability of the National Lung Screening Trial

**DOI:** 10.1001/jamanetworkopen.2026.8622

**Published:** 2026-04-27

**Authors:** Alison S. Rustagi, Marzieh Vali, Francis J. Graham, Emily Lum, Louise C. Walter, Amy L. Byers, Katherine J. Hoggatt, Beth E. Cohen, Salomeh Keyhani

**Affiliations:** 1 Center for Data to Discovery and Delivery Innovation (3DI), San Francisco Veterans Affairs Health Care System, San Francisco, California; 2 Division of General Internal Medicine, University of California, San Francisco; 3 Northern California Institute for Research and Education, San Francisco Veterans Affairs Health Care System; 4 Division of Geriatrics, University of California, San Francisco and San Francisco Veterans Affairs Medical Center; 5 Research Service, San Francisco Veterans Affairs Health Care System, San Francisco, California; 6 Department of Psychiatry and Behavioral Sciences, University of California, San Francisco,; 7 Weill Institute for Neurosciences, University of California, San Francisco

## Abstract

This cohort study evaluates all-cause mortality of National Lung Screening Trial participants compared with those of similar age and tobacco use in a national Veterans Health Administration cohort.

## Introduction

Lung cancer screening (LCS) reduced lung cancer death by 20% in the National Lung Screening Trial (NLST).^1^ NLST excluded those with life-limiting comorbidities. However, patients in routine care may be less healthy.^2^ Guidelines recommend screening those in good health, as only those who live beyond an averted cancer death benefit from screening.^3,4^ Direct comparisons are lacking between NLST participants and screening-eligible or screened patients in routine care. The Veterans Health Administration (VA) is a national pioneer in LCS and can address these knowledge gaps.^5^

We compared all-cause mortality of NLST participants with those of similar age and tobacco use in a national VA cohort.^6^ We hypothesized trial participants would have lower mortality than screening-eligible or screened patients in routine care and that health metrics could identify patients of similar health to trial participants.

## Methods

Full are in the eMethods in Supplement 1. Ethical approval for this study was provided by the University of California, San Francisco human research protection program. Reporting followed the Strengthening the Reporting of Observational Studies in Epidemiology (STROBE) reporting guidelines for cohort studies. We restricted 53 454 NLST participants to those randomized to control. We restricted 4503 VA cohort members^6^ to those meeting NLST eligibility (≥30 cigarette pack-years, current use or quit <15 years prior). Care Assessment Needs (CAN) score, a VA metric predicting mortality, and self-rated health and ability to climb stairs^4^ were captured at baseline. We restricted to ages 65 to 74 years, the range of overlap. Cohort enrollment occurred during 2020 to 2023 with follow-up through July 2025. Participants in the veteran cohort provided verbal informed consent. Participants in the NLST cohort provided written informed consent.

We compared 5-year all-cause mortality using Kaplan-Meier plots and Cox proportional hazards models between NLST controls and (1) cohort members, (2) those who received LCS, (3) cohort members stratified by CAN score, and (4) cohort members stratified by self-rated health and/or ability to climb stairs. Cohort analyses were weighted to reflect the national sampling design. Sensitivity analyses were restricted to cohort members who did not use cannabis (as the parent study examines cannabis’ health effects^6^) and who enrolled after January 1, 2022, after the early pandemic. *P* values were calculated using 2-sided log-rank tests, with *P* < .05 considered statistically significant. Analyses were completed in R version 4.5.2 (R Project for Statistical Computing).

## Results

Cohort members (732 individuals; 415 [57%] aged 65-70 years) were more likely male (642 patients [88%]) and less likely White (103 [14%] Black non-Hispanic; 33 [4.5%] not Black, not White, and non-Hispanic; and 569 [78%] White non-Hispanic) than NLST controls but had similar tobacco use (Table). Cohort screening prevalence was 25% (95% CI, 22%-28%) and did not vary significantly by favorable or unfavorable CAN score (<60: 25%; 95% CI, 19%-30% vs ≥60: 25%; 95% CI, 21%-29%) or favorable or unfavorable self-rated health and stair-climbing ability (26%; 95% CI, 22%-29% vs 18%; 95% CI, 10%-25%).

**Table.** Baseline Characteristics of National Lung Screening Trial (NLST) Controls (n = 7105) and Community-Dwelling Veterans in an Observational Cohort (n = 732)

Characteristic	No. (column %) [95% CI]	
	NLST controls (n = 7105)	Cohort (n = 732)^a^
Age, y		
65-70	5407 (76.1) [75.1-77.1]	415 (56.7) [53.0-60.3]
71-74	1698 (23.9) [22.9-24.9]	317 (43.3) [39.7-46.9]
Sex		
Female	2723 (38.3) [37.2-39.5]	90 (12.3) [10.0-14.9]
Male	4382 (61.7) [60.5-62.8]	642 (87.7) [85.1-89.9]
Race and ethnicity^b^		
Hispanic	112 (1.6) [1.3-1.9]	27 (3.7) [2.4-5.3]
Black and non-Hispanic	227 (3.2) [2.8-3.6]	103 (14.1) [11.6-16.8]
White and non-Hispanic	6470 (91.1) [90.4-91.7]	569 (77.7) [74.5-80.7]
Not Black, not White, and non-Hispanic	296 (4.2) [3.7-4.7]	33 (4.5) [3.1-6.2]
Marital status		
Other	2408 (33.9) [32.8-35.0]	405 (55.3) [51.6-58.9]
Married, partner, engaged	4697 (66.1) [64.9-67.2]	327 (44.7) [41.0-48.3]
Education		
Less than high school graduate	551 (7.8) [7.1-8.4]	43 (5.9) [4.3-7.8]
High school/some college degree	4177 (58.8) [57.6-59.9]	576 (78.7) [75.5-81.6]
Bachelor’s and beyond	2164 (30.5) [29.4-31.5]	112 (15.3) [12.8-18.1]
Refused/do not know	213 (3.0) [2.6-3.4]	1 (0.1) [0.003-0.8]
Tobacco cigarette use		
Pack-years, mean (SD)	61.2 (25.4)	61.2 (27.4)
Current smokers	2896 (40.8) [39.6-41.9]	374 (51.1) [47.4-54.7]
Quit duration, for former smokers, mean (SD)	8.3 (4.8)	6.9 (4.9)

^a^ Cohort members were VA primary care patients with a 30 or more tobacco pack-year history and current smokers or quit less than 15 years before study enrollment. Median (IQR) follow-up was 79 (74-83) months for NLST controls, and 53 (44-58) months for cohort members.

^b^ For NLST participants, race and ethnicity was self-reported at baseline via questionnaire. For cohort members, race and ethnicity were self-reported in the electronic medical record.

Over 5 years, 685 of 7105 individuals in the NLST control group (9.7%) and 136 of 732 cohort members (24.3%) died. Cohort members experienced 3-fold higher mortality than NLST controls (hazard ratio [HR], 2.98; 95% CI, 2.48-3.59) (Figure, A), as did those screened for lung cancer (HR, 3.08; 95% CI, 2.17-4.37) (Figure, B). Findings were unchanged in sensitivity analyses. At a CAN of 40 or greater (659 individuals [90.4%]), cohort members experienced higher all-cause mortality than NLST controls (Figure, C), as did those with fair or poor self-rated health and difficulty climbing stairs (Figure, D).

**Figure.**
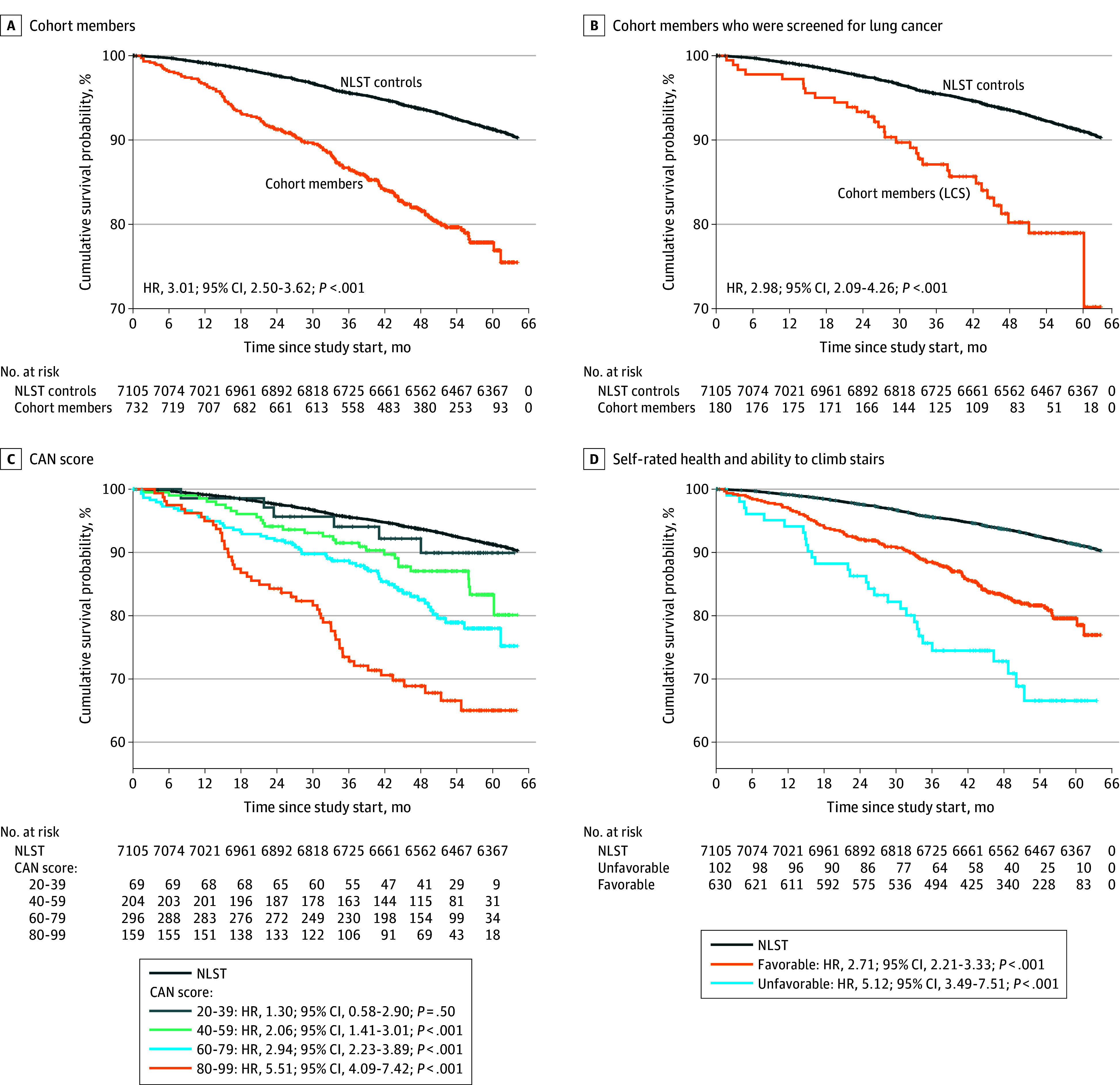
Cumulative Survival Among National Lung Screening Trial (NLST) Control Participants (n = 7105) and Community-Dwelling Veterans in an Observational Cohort (n = 732) Cohort members were Veterans Affairs (VA) primary care patients with a 30 or more tobacco pack-year history and current smokers or quit less than 15 years before study enrollment. Covariate adjustment was not performed as the study sought to understand population-level differences between NLST participants vs those in routine care of similar age and tobacco cigarette use. CAN score (0-100) is automated, objective, updated weekly, and widely integrated into VA clinical dashboards. No deaths were observed among cohort members with CAN scores of 0 through 19 (3 individuals). For self-rated health and ability to climb stairs, favorable health was defined as self-rated health good, very good, or excellent with or without difficulty climbing stairs. Unfavorable health was defined as fair or poor self-rated health and limited ability to climb stairs. CAN indicates Care Assessment Needs; HR, hazard ratio.

## Discussion

In this study, older veterans eligible for or receiving LCS had a greater risk of mortality than NLST participants of similar age and tobacco history. These findings suggest that the lung cancer mortality reduction from LCS may be smaller in routine care than in the NLST (20%).

To maximize benefits, clinicians could promote screening patients with similar mortality as NLST participants (ie, <10% 5-year mortality) via shared decision-making.^3^ Veterans with a CAN of 40 or higher or with fair or poor self-rated health and limitations in climbing stairs experienced higher mortality than NLST controls and may be unlikely to benefit. Screening is not benign and can cause harm from diagnostic procedures or overdiagnosis. Comorbidities should be optimized for patients who pursue LCS.

Study strengths include using a national cohort with known sampling weights, tobacco history, health metrics, and screening use. Limitations include that results may not generalize to non-VA settings, other ages, or VA screening implementation after 2023. Cause of death was unavailable. Programs to expand LCS may not achieve their full potential without concerted efforts to screen those most likely to benefit.

## Notes

Supplement 1. eMethods. eReferences.

Supplement 2. Data Sharing Statement
