# Supplementary material for: Mortality and Generalizability of the National Lung Screening Trial
Source: JAMA Netw Open. 2026 Apr 27;9(4):e268622. doi: 10.1001/jamanetworkopen.2026.8622 (PMC13122399; doi:10.1001/jamanetworkopen.2026.8622)
Supplement: Supplement 1. — eMethods. eReferences. [file jamanetwopen-e268622-s001.pdf]

Supplemental Online Content

Rustagi AS, Vali M, Graham FJ, et al. Mortality and generalizability of the National Lung Screening Trial. *JAMA Netw Open*. 2026;9(4):e268622.
doi:10.1001/jamanetworkopen.2026.8622

eMethods.

eReferences.

This supplemental material has been provided by the authors to give readers additional information about their work.

© 2026 Rustagi AS et al. *JAMA Network Open*.

eMethods

Ethical approval for this study was provided by the University of California-San Francisco Human Research Protection Program (#18-26608, #23-39731). Reporting followed the STROBE reporting requirements for cohort studies.

All analyses were conducted in R version 4.5.2 (2025-10-31 ucrt) using the following libraries: RODBC, sqldf, tidyverse, sqldf, gmodels, glue, pROC, LogicReg, DescTools, Hmisc, descr, survey, ggplot2, remotes, PSweight, survival, survminer, gtsummary, reshape2, nlme, gee, geepack, ggeffects, parallel, MatchIt, lcrisks, gt, gridExtra.

We utilized data from (1) NLST control arm participants[1] (referred to as "NLST controls" hereafter) and (2) veterans enrolled in the prospective observational Cannabis and Aging Cohort (referred to as "cohort members" hereafter). We applied the following exclusions to generate comparison groups that were similar in terms of age and tobacco cigarette history.

NLST Cohort

The NLST enrolled healthy individuals during 2002-2004 who were ages 55-74 with a 30+ year tobacco cigarette pack-year history, who currently smoked or quit <15 years prior. For this analysis, we restricted NLST participants to those who were (1) randomized to the control arm, as the intervention had a measurable impact on all-cause mortality, largely driven by lower lung cancer mortality,[1] and (2) ages 65-74 years at baseline to promote comparability with the cohort, which required a minimum age at enrollment of 65 years. This project received approval and data access from the Cancer Data Access System at the National Cancer Institute (CDAS Project # NLST-1236). The NLST was registered under ClinicalTrials.gov registration number NCT00047385.

© 2026 Rustagi AS et al. *JAMA Network Open*.

Veteran Cohort Members

We used the Cannabis and Aging Cohort data to define our veteran cohort. This study enrolled n=4,503 patients ages 65-84 years with at least one VA primary care visit in the two years prior to cohort entry.[2]

The study enrolled participants from February 5, 2020 to August 29, 2023, with a robust response rate of 50.5%. The study excluded those enrolled in hospice/palliative care, receiving active cancer treatment, unable to participate in a phone interview, or residing in a nursing home, or those with dementia or severe uncontrolled mental health conditions such as active suicidal ideation. Informed consent was obtained before study enrollment. The cohort oversampled women and those who used cannabis; sampling weights based on age, sex, race, ethnicity, U.S. census region, and the presence of cannabis-related terms in medical notes were known relative to the national population of eligible patients in VA primary care (n=2,590,235).

For this analysis, we restricted to cohort members who met the NLST's tobacco cigarette pack-year eligibility criteria. Tobacco cigarette pack-year history was ascertained for all cohort members in a one-on-one interview. These eligibility criteria informed the 2013 U.S. Preventive Services Task Force (USPSTF) screening eligibility guidelines (ages 55-80 years, 30+ year tobacco cigarette pack-year history, currently smoking or quit date <15 years prior) and thus all included cohort members were LCS-eligible by 2013 guidelines.[3] We also restricted the cohort to those 65-74 years, as the NLST imposed a maximum age at enrollment of 74 years.

Lung Cancer Screening

We used a published algorithm[4] to identify lung cancer screening tests among cohort members in the five years before cohort entry; that is, LDCTs ordered for the purpose of screening asymptomatic patients for lung cancer. Individuals who receive chest imaging for the purpose of screening are more healthy than

© 2026 Rustagi AS et al. *JAMA Network Open*.

symptomatic patients because, by definition, screening tests are ordered in patients with no signs/symptoms of disease.

Algorithm to identify chest imaging performed for lung cancer screening

Briefly, the algorithm captured outpatient LDCTs in the five years prior to study enrollment using administrative codes (CPT codes 71250, G0297, S8023, or 71271) and has been described in detail previously.[4] We then examined free text terms in radiology orders indicating LCS ("screen," "screening," "LCS") in Clinical History or Reason for Exam fields. We excluded imaging studies ordered in inpatient or emergency room encounters and those with free text terms indicating the test was prompted by signs/symptoms of disease (e.g., "cough," "wt loss," "chest pain"). This algorithm was validated against the gold standard of manual chart review of medical notes and found to have a 95% positive predictive value for a true screening exam (i.e., ordered for lung cancer screening in an asymptomatic patient with no signs or symptoms of disease).[4]

Health metrics

To assess whether national guidelines to screen healthy individuals are followed in practice, we used two measures of health.

First, we characterized patients by Care Assessment Needs (CAN) score at study enrollment. CAN score was missing for 4 of 732 cohort members (<1%). CAN score is a percentile ranging from 0 to 100, and is validated to predict hospitalization and all-cause mortality among VA primary care patients.[10] At a CAN score of 60, a patient's predicted all-cause mortality over one year (1.3%)[10] is similar than that observed among NLST control participants over the duration of the trial (1395/100,000 person-years).[8] Predicted mortality increases exponentially at CAN scores above 60.[10] Thus we chose *a priori* to use CAN≥60 as a threshold of favorable/unfavorable health relevant to LCS. In exploratory analyses, we stratified by CAN score (0-19, 20-39, 40-59, 60-79, 80-99).

© 2026 Rustagi AS et al. *JAMA Network Open*.

Second, we characterized health using self-rated health and ability to climb stairs, which were ascertained at cohort enrollment. Self-rated health predicts all-cause mortality more accurately than a 30-item objective measure of health,[5,6] and stair climbing is a common pre-operative measure of cardiovascular fitness.[7] These metrics have been proposed as a pre-screening health metric relevant to LCS,[8] in part because the pivotal European trial of LCS (NELSON) excluded those with fair/poor self-rated health who were limited a lot in climbing stairs.[9] Thus an 'unfavorable' rating on this metric was defined as self-rated health of fair/poor and being limited a lot in climbing stairs. A 'favorable' rating on this metric was defined as self-rated health of good/very good/excellent and/or not limited a lot in climbing stairs.

Primary Outcome

Our primary outcome was all-cause mortality in the five years after study enrollment using vital status and death date from the National Death Index (NLST), or in VA's Corporate Data Warehouse (cohort members). Individuals were followed from the time of randomization (NLST controls) or baseline interview (cohort members) until death, administrative end date (July 21, 2025), or 5 years had elapsed, whichever came first. We compared NLST controls versus: 1) all cohort members, 2) those who received LCS, 3) cohort members stratified by CAN score, and 4) cohort members stratified by self-rated health and ability to climb stairs (favorable/unfavorable as defined above).

Statistical analysis

We calculated 5-year cumulative all-cause mortality to generate Kaplan Meier survival plots (1 – cumulative mortality) for the NLST and the veteran cohort, and compared all-cause mortality using the log-rank test. We used Cox proportional hazards models to quantify relative differences in 5-year all-cause mortality using point and interval estimates of the hazard ratio. We did not adjust for covariates as our primary research question was to understand actual population-level differences between the overall

© 2026 Rustagi AS et al. *JAMA Network Open*.

health of those in the NLST versus those in community settings of similar age and tobacco cigarette use. All analyses applied person-level analytic weights to provide estimates representative of the larger population of VA patients (the target population from which the sample was drawn).

Sensitivity Analyses

First, to examine whether cannabis use impacted our point estimates, we restricted to cohort members who denied cannabis use in the 30 days prior to cohort enrollment (n=468). Second, we excluded veterans who enrolled in the cohort during 2020 and 2021 to remove the contribution of the early COVID-19 pandemic on excess mortality. To understand how LCS is used across the full spectrum of health, we examined LCS prevalence across different definitions of optimal/sub-optimal health (self-rated health, ability to climb stairs, CAN score). Statistical analyses were conducted in R, version R-4.4.1.

© 2026 Rustagi AS et al. *JAMA Network Open*.

eReferences

1. The National Lung Screening Trial Research Team. Reduced Lung-Cancer Mortality with Low-Dose Computed Tomographic Screening. *N Engl J Med*. 2011;365(5):395-409. doi:10.1056/NEJMoa1102873

2. Pravosud V, Lum EN, Vali M, et al. The VA Cannabis and Aging Cohort: Correlates of Cannabis use among Older Adults. *JAMA Netw Open*. Published online in press 2025.

3. Moyer VA, U.S. Preventive Services Task Force. Screening for lung cancer: U.S. Preventive Services Task Force recommendation statement. *Ann Intern Med*. 2014;160(5):330-338. doi:10.7326/M13-2771

4. Rustagi AS, Vali M, Graham FJ, Lum EN, Slatore CG, Keyhani S. A Novel Automated Algorithm to Identify Lung Cancer Screening from Free Text of Radiology Orders. *J Gen Intern Med*. Published online February 25, 2025. doi:10.1007/s11606-025-09429-2

5. Wuorela M, Lavonius S, Salminen M, Vahlberg T, Viitanen M, Viikari L. Self-rated health and objective health status as predictors of all-cause mortality among older people: a prospective study with a 5-, 10-, and 27-year follow-up. *BMC Geriatr*. 2020;20(1):120. doi:10.1186/s12877-020-01516-9

6. DeSalvo KB, Bloser N, Reynolds K, He J, Muntner P. Mortality prediction with a single general self-rated health question: A meta-analysis. *J Gen Intern Med*. 2006;21(3):267-275. doi:10.1111/j.1525-1497.2005.00291.x

7. Smilowitz NR, Berger JS. Perioperative Cardiovascular Risk Assessment and Management for Noncardiac Surgery: A Review. *JAMA*. 2020;324(3):279-290. doi:10.1001/jama.2020.7840

8. Rustagi AS, Slatore CG, Keyhani S. Self-Rated Health and Ability to Climb Stairs: A Pragmatic Health Assessment Before Lung Cancer Screening. *Ann Intern Med*. 2023;176(4):568-571. doi:10.7326/M22-3598

9. de Koning HJ, van der Aalst CM, de Jong PA, et al. Reduced Lung-Cancer Mortality with Volume CT Screening in a Randomized Trial. *N Engl J Med*. 2020;382(6):503-513. doi:10.1056/NEJMoa1911793

10. Wang L, Porter B, Maynard C, et al. Predicting risk of hospitalization or death among patients receiving primary care in the Veterans Health Administration. *Med Care*. 2013;51(4):368-373. doi:10.1097/MLR.0b013e31827da95a

© 2026 Rustagi AS et al. *JAMA Network Open*.
