# Supplementary material for: Mortality and Generalizability of the National Lung Screening Trial
Source: JAMA Netw Open. 2026 Apr 27;9(4):e268622. doi: 10.1001/jamanetworkopen.2026.8622 (PMC13122399; doi:10.1001/jamanetworkopen.2026.8622)
Supplement: Supplement 2. — Data Sharing Statement [file jamanetwopen-e268622-s002.pdf]

Data Sharing Statement

Rustagi. Mortality and Generalizability of the National Lung Screening Trial. *JAMA Netw Open*. Published April 27, 2026. doi:10.1001/jamanetworkopen.2026.8622

Data
Data available: Yes
Data types: Deidentified participant data
How to access data: Access to the NLST data is available by request through the Cancer Data Access System at https://cdas.cancer.gov/. Access to the VA data is possible with adherence to VA policy and procedures. De-identified data may be made available upon reasonable request to the Principal Investigator, Dr. Alison S. Rustagi.
When available: With publication

Supporting Documents
Document types: None

Additional Information
Who can access the data: Access to the NLST data is available by request through the Cancer Data Access System at https://cdas.cancer.gov/. Access to the VA data is possible with adherence to VA policy and procedures. De-identified data may be made available upon reasonable request to the Principal Investigator, Dr. Alison S. Rustagi.
Types of analyses: NA
Mechanisms of data availability: Access to the NLST data is available by request through the Cancer Data Access System at https://cdas.cancer.gov/. Access to the VA data is possible with adherence to VA policy and procedures. De-identified data may be made available upon reasonable request to the Principal Investigator, Dr. Alison S. Rustagi.
